# The student migration transition: an empirical investigation into the nexus between development and international student migration

**DOI:** 10.1186/s40878-023-00329-0

**Published:** 2023-04-03

**Authors:** Tijmen Weber, Christof Van Mol

**Affiliations:** 1 HAN University of Applied Science, Arnhem, The Netherlands; 2 Radboud University, Nijmegen, The Netherlands; 3 Tilburg University, Tilburg, The Netherlands

**Keywords:** International student mobility, Human development, Migration transition theories, Gravity models

## Abstract

In this paper, we analyze the relationship between development and outgoing international student mobility (ISM) for the years 2003–2018 using data from UNESCO. Starting from migration transition theory, we expect that development and outgoing migration follows an inverted U-shape due to changes in capabilities and aspirations of populations. As predicted, we find that outgoing ISM also follows this pattern. Probing deeper into this finding, we investigated whether students from countries of different levels of development favor different destination countries, focusing on destination countries’ academic ranking, GDP per capita, and linguistic and colonial ties. We find that these destination country characteristics indeed have different effects for students from origin countries with different stages of development, and that these effects cannot simply be reduced to a dichotomy between developed/developing countries. Together, the findings highlight the nonlinearity of ISM processes. In turn this opens up new avenues of research regarding the diversity of international student populations.

## Introduction

Over the last two decades, the number of international students enrolled in tertiary education has been increasing at a steady rate, both in absolute and relative terms. For example, the number of international students increased by 186% between 1998 and 2018, while the total number of students enrolled in higher education increased by 152%.^1^ This growth in international student migration coincides with trends concerning the internationalization of higher education, which have been gaining prominence over the last few decades, as well as societal developments such as the rise of the middle class in many non-western countries such as China and India, whereby international student migration (ISM) can form a strategy of social distinction. Despite these trends, there is still relatively little theoretically grounded empirical research on the determinants of international student migration at a macro-level compared to studies that focus on international students’ individual-level decision-making processes. Furthermore, only a handful of papers on the determinants of international student migration took the development level of host and origin countries into account, even though the link between development and migration has been extensively discussed in other areas of migration studies (Geiger & Pécoud, 2013). Consequently, in this paper we aim to expand our scientific knowledge on the determinants of international student migration, by exploring how differences in international student migration flows can be explained by differences in development between sending and receiving countries, building further upon insights from the literature on the migration-development nexus.

Our theoretical starting point are migration transition theories (De Haas, 2010; De Haas et al., 2019, 2020; Skeldon, 2012; Zelinsky, 1971). These theories allow us to consider patterns of international student migration as ‘an intrinsic part of broader processes of development and social transformation associated with processes of modernization and industrialization’ (De Haas et al., 2020: 56). Transition theories particularly predict that the relationship between development and migration ‘is complex and fundamentally non-linear’ (De Haas et al., 2020: 56), generally indicating that emigration follows an inverted U-shape pattern. Initially, development boosts emigration, but once countries reach a certain level of development, countries tend to transform from net emigration into net immigration countries. In this paper, we empirically assess whether international student migration flows follow a similar pattern.

In this study, we focus on degree mobility: international students who cross a border with the intent of completing an entire degree outside of their country of origin. We rely on UNESCO data^2^ on global student migration flows between 2003 and 2018. In the first part of this study, the hypothesis of a student migration transition is tested by investigating whether the relationship between the Human Development Index and outgoing and incoming international student migration flows follows the predicted inverted U-shape pattern. In the second part, we conduct a more explorative analysis where we investigate the effect of two host country characteristics (academic ranking and GDP), and two dyadic characteristics (common official language and colonial past) on incoming international students both between and within host countries over time. Most importantly, we explore whether these characteristics have different effects for students from countries of different stages of development.

Our analysis makes three major contributions to the scholarly literature. First, we empirically test whether a well-established set of theories in demography and migration studies, namely transition theories, is also relevant for explaining the direction of global international student migration flows. Such theoretically informed approach remains an exception rather than the norm in macro-level studies on international student migration but has great potential to advance both our theoretical and empirical understanding of international student migration. Second, we unravel the complexity and diversity in the determinants of international student migration. We particularly move beyond approaches that consider international students to be a homogenous group by analyzing how the importance of host country characteristics might significantly vary across countries, taking different levels of development into account. Finally, by comparing both differences between and within countries we produce different estimates which is especially important, because prior studies that looked at these trends either used static measures (e.g. Börjesson, 2017; Didisse et al., 2018; Siekierski et al., 2018) or did not analyze recent flows (e.g. Beine et al., 2014; Caruso & de Wit, 2015; Van Bouwel & Veugelers, 2013; Wei, 2013). In contrast, our analyses enable us to also study the effect of a country characteristic as it changes over time while mitigating differences between countries.

## Literature review

### Migration transition theories and international student migration

Migration Transition Theories are a set of theories that tend to explain why—in contrast to policy discourses which often argue the opposite—development tends to increase emigration (see for example (Zelinsky, 1971; De Haas, 2010, a; Skeldon, 2012; De Haas et al., 2020). Specifically, migration transition theories indicate that the relationship between developmental processes and emigration rates follows an inverted U-shape. As countries become more developed, they will experience an increase in emigration, but this increase will reach a saturation point: once countries reach a certain level of development, it is hypothesized they would become net immigration countries. De Haas (2020) and Carling (2002), Carling and Schewel (2018) provided explanations for the micro-level mechanisms behind the macro-level processes transition theories describe by linking developmental processes to a capabilities and aspirations framework. Shortly put, capabilities refer to human, social, and financial capital that is needed to overcome barriers of migration. It is expected that development increases the capabilities of individuals and households, for example through income growth, improved education, and improved communication and transport links, making migration more accessible to larger parts of the population (De Haas et al., 2020), as migration is a costly enterprise. At the same time,’aspirations are a function of people’s general life aspirations and perceived geographical opportunity structures’ (De Haas et al., 2020: 62). Hence, if people have certain aspirations they cannot realize at home, migration aspirations might increase. As such, aspirations are likely affected by a country’s level of development. Thus, when poor countries develop, migration aspirations might increase as individuals might become more aware of opportunities and conditions in other places. However, once the development reaches a certain point these aspirations likely start to drop, as opportunity gaps with destination countries decrease (De Haas et al., 2020). While the capabilities-aspirations framework focuses on migration in general, we believe it is also applicable to international students. As Van Mol (2014) argued, from a theoretical perspective international students can be considered to be a specific type of migrants, whose migration trajectory is underpinned by migration dynamics that are in many respects similar to more ‘classical’ forms of international migration, such as labor migration. Furthermore, also in terms of temporality and possible return to the country of origin after graduation ISM resonates with other forms of international migration such as retirement migration or seasonal migration. Given that student migration is often driven by similar macro-, meso- and micro-level factors compared to other international migration forms (see e.g. Van Mol et al., 2023), it can be hypothesized the same mechanisms are at play for international student mobility: when development in countries in the global south increases, the aspirations and capabilities of young adults to engage in an international study might concomitantly increase, leading to higher outgoing student mobility rates up to a certain development threshold.

We argue that compared to the popular push–pull models in the ISM-literature, transition theories are a useful starting point for analyzing the relationship between development and international student migration, as they do not have the drawback of considering migration as a pre-determined set of linear choices and preferences (Lipura & Collins, 2020). Indeed, push–pull models do not take into account the fluid nature of contexts, and how changing conditions might affect the decision to move to certain destination countries for studying—or not. We also advance our understanding of international student migration by investigating two host country characteristics: ranking and GDP, and two dyadic country characteristics: linguistic and colonies ties, analyzing how these differ for students from countries of higher or lower levels of development. In the sections below, it will be explained how these three characteristics tie in with the capabilities and aspirations framework that is at the heart of migration transition theory as discussed by De Haas et al. (2020).

### Capabilities in the context of international student mobility

A first point for consideration is prior education: Naidoo (2007) indicated that to become an international student in higher education generally requires an individual to have finished relevant pre-education. However, educational attainment levels of a given population also increase with development: global inequalities regarding the years of schooling individuals obtain have been reported extensively (see e.g. Barro & Lee, 2013). Consequently, it can be expected that when development increases, the number of students accessing higher education increases, and the capabilities of young people consequently increase, whereby embarking on a foreign degree might become an option.

Apart from a relevant pre-education, students also need other capabilities in order to be able to study abroad. Not only do they need to bear the costs of travel and visa applications, tuition fees and cost of living can also be substantially higher than what they are used to in their country of origin. For example, tuition fees are related to decision-making processes for entering higher education, both domestically (see e.g. Wilkins et al., 2013) as well as internationally (see Naidoo, 2007). This can also be seen in other contexts such as a study done by Perkins and Neumayer (2014), who found that ranking was a stronger factor for students from highly developed countries than for developing countries. In part this can be explained by the fact that countries with higher ranked institutions tend to have both higher tuition fees and higher costs of living, which makes them not easily accessible to all international students. Because of this, it is expected that countries with higher GDPs and higher rankings host more students from higher developed countries as they more often have the means to afford living in more expensive destinations. However, it should also not be forgotten that countries can influence what capabilities are needed. For example, in the EU/EEA tuition fees are often lower for students from other EU/EEA countries, and VISA requirements are also more lenient for them. At the same time, countries and institutions can award scholarships in order to aid students from lower developed countries. The capabilities needed to become an international student are therefore not just dependent on someone’s origin country, but also on the origin country’s relation to destination countries.

### Aspirations in the context of international student mobility

De Haas (2021) distinguishes two types of aspirations that can motivate individuals to become mobile: instrumental and intrinsic. Instrumental aspirations refer to migration as a means to an end such as income maximization, or in the case of students, access to better education and future employment opportunities. Conversely, intrinsic aspirations refer to value that is attached to the migration experience itself such as enjoying new cultures or migrating as a rite of passage.

Access to better education is a particularly important type of instrumental aspiration since not all countries are capable of providing a degree of high value to all students because the institutions are not of high quality, or because domestic higher education systems do not dispose of sufficient capacity. For example, East Asian students tend to study abroad because a foreign degree gives more prestige or because they could not get into local institutions (Brooks & Waters, 2011). According to credentialism theory (Bills & Brown, 2011), a degree signals that someone has certain desirable qualities. It is therefore not necessarily the quality of the education as such that matters, but rather the perceived quality. Because of this, international students are more likely to favor countries that are perceived to provide better quality degrees (Cebolla-Boado et al., 2018; Van Bouwel & Veugelers, 2013). Kritz (2016) also indicated that countries that have less tertiary education supply are more likely to have higher outbound migration ratios. However, students coming from highly developed countries often already have access to high quality education so they might have more intrinsic aspirations for migrating in addition to instrumental aspirations. Previous research seems to partially confirm these differences in aspirations. For example, Kondakci (2011) found that for international students in Turkey, students from Western countries put more emphasis on the desire to experience Turkish culture and improve their intercultural understanding, whereas students from Eastern European and developing countries put more emphasis on economic and academic rationales. Wei (2013), on the contrary, found that when considering a developed country as a destination, students from developed countries place more importance on academic factors, while students from developing countries valued economic factors more. However, an alternative explanation is that many of the high-ranked higher education institutions are situated in Anglo-Saxon countries in which international student mobility has increasingly become marketized which could result in more active recruitment and marketing (Börjesson, 2017; Findlay et al., 2017).

Regarding GDP per capita, it has been found that richer countries tend to be more attractive for students to study in (Caruso & de Wit, 2015; Dreher & Poutvaara, 2005; Wei, 2013). At the micro-level, this might translate in both intrinsic and instrumental motivations. For instance, richer countries have more amenities and might also offer better employment opportunities. However, at the same time richer countries can also invest more in their education and spend more money on recruiting international students, and often there is an incentive to recruit international students in order to fill gaps in the labor market (Levatino et al., 2018). Although studying in richer countries generally also means higher costs of living, Caruso and de Wit (2015) found that this does not deter students from moving. However, Wei (2013) suggests that for students from developing countries cost of living outweighs other factors, and de Wit and Altbach (2020) suggest that, among other things, lower costs makes countries like China, Malaysia and India attractive to neighboring countries and Africa. Therefore, it is expected that GDP has a positive effect on the number of international students in a host country, but that this effect is stronger for students from more highly developed countries.

Finally, we investigate the effect of linguistic ties and colonial history between country pairs when it comes to international student enrollment. Such relationships have been identified by several scholars (e.g. Baláž et al., 2018; Barnett et al., 2016; Chankseliani, 2016; Kondakci et al., 2018; Mulvey, 2021; Ovchinnikova et al., 2022; Vögtle & Windzio, 2016), but these studies did not take differences in development of countries into account when analyzing this link. Many higher education systems in top-destination countries (e.g. France, Germany, Russia) do not always offer many higher education programs in English and therefore might be inaccessible to students not speaking the local language. If countries share a common language, then such barriers would be easier to overcome. Former colonial ties also play a role in structuring global ISM flows. For example, Portugal subsidizes places for students from former Portuguese colonies (Sin et al., 2019), France has looser VISA requirements for students from French-speaking countries (Highman & de Gayardon, 2022), and Russia allows VISA free enrollment for many of its neighboring countries (Minaeva & Prostakov, 2022). Indeed, Börjesson (2017) uncovered three main poles in the flows of international students: a Pacific/Market pole constituted by Anglophone countries, a proximity/linguistic pole constituted by Central European countries, and colonial pole constituted by France, Spain, and Portugal.

In line with these previous studies, we thus expect that both linguistic and colonial ties are related to higher enrollments. However, in contrast to previous studies, we also expect that these effects are stronger for lower developed countries because highly developed countries are more likely to host international students from countries in their sphere of influence than the other way around. In part this could be because migration tends to be costlier for students from lower developed countries so it might be more attractive to study in countries that have linguistic and colonial ties as it lowers the ‘migration costs’ associated with moving to another country. Students from higher developed countries might in contrast have more capabilities in terms of finances, English-language skills, or cultural capital, providing them more opportunities in terms of deciding where to enroll.

A shortcoming of previous ISM studies on the subject is that they only considered development as a dichotomy between developed and developing countries, but as migration transition theory states, the relationship between development and migration happens in several stages. For this reason, this paper divides countries in five equally sized quintiles similar to De Haas, (2010) in order to better capture the diversity of different stages of development and how this affects the choice of destination country. In sum, in this paper we empirically test whether international student migration follows the same dynamics as predicted by migration transition theories, which focus on general migration dynamics.

## Research design

### International student mobility and human development

The data regarding (international) student populations was retrieved from the UNESCO Educational Statistics database,^3^ which provides data on international student flows between countries. Not all countries were included; notably all the microstates were excluded as well as some other countries which had missing data on one or several of the relevant variables. For testing the student migration transition model, the remaining origin countries accounted for 98.9% of the world’s population while the host countries accounted for 78.1%^4^ in 2018. To measure the development level of a country, the Human Development Index (HDI) was used which was created to measure a country’s development (Ul Haq 1995). This is index combines three dimensions of the well-being of a country: Education, Life Expectancy, and GDP per capita. These dimensions are combined into a single index ranging from 0 to 1.

Testing whether we could find the inverted U-shape predicted by migration transition theory was done by plotting the HDI index against the total number of outgoing and incoming international students as a percentage of a country’s population aged 15–24 for the year 2018.^5^ It was decided to use the youth population because most international students consist of young people and using the total population would present biased results because of differences in the demographic make-up of countries (some countries have relatively more young people than other countries) which is why the youth population is a better proxy. Further, we chose this measure instead of the total student population to better represent the capabilities dimension, i.e., in lower developed countries it would be expected that there are fewer outgoing international students partially because there are fewer students in general. The relationship between the variables is analyzed using a non-parametric regression based on a loess curve. This technique makes it possible to reveal non-linear relationships which best fit the data in a scatterplot (Jacoby, 2000). In turn this makes it possible to see whether the predicted inverted U-shape is indeed the best fitting pattern in the data.

### Differences in characteristics of destination countries

In order to analyze host country characteristics that attract students and how these differ between students coming from countries of different stages of development a gravity model is estimated. In short, a gravity model in this context assumes that the number of students from country A from country B is a function of the total student population in country A and B, the distance between the countries, and other relevant variables which can make it attractive or unattractive to migrate. This model has been widely used in the literature (see e.g. Bessey, 2012; Naidoo, 2007; Van Bouwel & Veugelers, 2013).

### Data

For estimating differences between destinations countries, more countries had to be excluded because of missing data on one or more of the variables though the origin countries still account for 97.4% of the world population while the host countries account for 66.5%.^6^ However, the countries that hosted the most students did have data available. For example, in 2017, the missing host countries only accounted for 6.2% of the total number of hosted international students. Although not all of the country pairs were in the data, the dataset still contained 98.2% of the possible country pairs (given the host and origin countries that are available). The final dataset includes data from 2003 until 2018 and focuses on 145 origin countries and 119 host countries, comprising 16,058 country pairs, and a total of 148,463 individual observations. A complete list of host and origin countries used in these analyses can be found in Appendix 1. We focus on the period 2003–2018 as our variable which measures ranking is available from 2003 onwards, and at the time of writing, 2018 was the most recent available data in the UNESCO dataset.

### (International) student populations

The dependent variable ${\text{Y}}_{{{\text{ijt}}}}$ in the regression model is the number of international students living in a host country (i) from an origin country (j) at a certain time point (t). As is standard in gravity models of this kind, the total student population of the host country and the total population aged 15-24 of the origin country were included as so-called exposure variables.^7^ Unfortunately, there were many missing values on the student population variable, so it was imputed using simple regression analysis with time as the independent variable except when the R-squared was lower than 0.7 and/or the number of observations was smaller than 4. For the host countries 5% of the variable consisted of imputed values. In the regression analyses, we included a dummy variable to indicate whether a value was imputed or not. All of this data was retrieved from UNESCO.

### Independent variables

The first country characteristic is academic ranking, which was measured using the Academic Ranking of World Universities^8^ (also known as the Shanghai Ranking) which was used by van Bouwel and Veugelers (2013) as well. However, while van Bouwel and Veugelers (2013) simply counted the number of institutions for each country in the top 500 list, we instead made use of the standardized sum score that each institution is assigned each year. We argue that this is better because a higher score means that a higher education institution is higher up on the list and is therefore more prestigious, a fact that is overlooked when simply counting the number of institutions. These scores do not extend past 100 on the list, but fortunately they follow a very predictable logarithmic pattern so the scores could be extrapolated using simple regression analysis (with the minimum score capped at 0). Ranking for each country is then measured by calculating the sum of the scores for all the institutions in a country in a certain year. The second country characteristic is GDP per capita for a country in a certain year adjusted for purchasing power parity (PPP) which was retrieved from the World Bank.^9^ Data regarding common official language and past historical ties were provided by the CEPII institute.^10^ Finally, we added two control variables namely distance between countries and whether countries share a border; both of these were also taken from the CEPII institute.

### Analytic strategy

For the second part of the paper, we estimated regression models using a negative binomial distribution. While Silva and Tenreyro (2006) recommend the use of a Poisson distribution, this method leads to biased results if there is overdispersion in the dependent variable, which was the case for the data in this study. In situations like these, a negative binomial distribution produces better results (Rönkkö et al., 2022). Since the negative binomial distribution estimates the logit of the dependent variable, we also log-transformed the exposure variables (total student population of the host country, and population aged 15-24 of the origin country). The necessity of this can be demonstrated algebraically. Consider the case of $\mu_{x}$ representing the parameter estimate of the dependent variable. A simple regression with a log transformed dependent variable would be expressed as:

$$\log \mu_{x} = \beta_{0} + \beta_{1x}$$

However, this method is flawed because it assumes that $\mu_{x}$ takes place in the same context for each country. Since every country has a differently sized pool of people to draw from, the count must be divided by an exposure variable $z_{x}$ (in our case the total number of students in the host country and the population aged 15–24 in the origin country):

$$\log \left( {\frac{{\mu_{x} }}{{z_{x} }}} \right) = \beta_{0} + \beta_{1x}$$

Which in turn can be rearranged as:

$$\log \mu_{x} = \log z_{x} + \beta_{0} + \beta_{1x}$$

Because the nature of the data is hierarchical, i.e. observations per year are not independent but nested in countries, we employed a multilevel regression which produces better estimates if the data has this structure (Hox et al., 2005). Specifically, we estimate a three-level cross-classified model by including three random intercepts in its three levels. Level 3 is at the country level and includes a random intercept for both the host country and the origin country. Level 2 is at the country pair level and includes a random intercept for country pair nested in the host country, and a random intercept for the country pair nested in the origin country. Level 1 is the number of international students in a host country from a certain origin country in a certain year.

In order to test differences between countries with different levels of development, the data was split up into five groups (quintiles) based on the HDI, ranging from lowest to highest. The model was then run a total of six times, once for each quintile and once for the total dataset. These quintiles were calculated seperately for each year in order to take into account the fact that, overall, the HDI of countries has been increasing. This does mean that countries can be in more than one quintile over this time period which reflects their increase or decrease in HDI. For example, Malaysia started in the “medium category” in 2003 but moved up to the “high” category in 2008. It is also for this reason that the total number of origin countries for the five quintiles together exceeds 145. Prior to presenting the results, the models were checked for multicollinearity but none was found.

A final consideration is how to effectively estimate the effects of variables with such complex units such as countries. To tackle this we enhance the traditional gravity model by estimating it as a between-within model (Neuhaus & Kalbfleisch, 1998). Previous macro studies have only compared differences on variables between countries but this can lead to omitted variable bias as unmeasured characteristics can muddle the true effect of an indicator. The strength of a between-within model is that it is able to estimate both differences of an indicator between countries, and crucially, estimate how changes of an indicator within countries over time affect international student migration. This gives a better indication of how the change of an indicator leads to a change in the number of international students in a country. Specifically, a between-within computes two variables for each relevant indicator: the mean of the indicator over the whole time period for a certain country, and the difference from the mean in a certain year for a certain country. For example, the host country indicator GDP per capita would compute a variable “mean GDP” for each of the host countries over the years 2003–2018. The second variable measures how much lower or higher the GDP of a country is in a certain year relative to the country’s mean.

The partial specification (control variables are not included) for this three-level cross-classified between-within model can be expressed as such:

$${\text{Y}}_{{{\text{tij}}}} = \updelta_{000} + \updelta_{001} \overline{X}_{i} + \updelta_{001} \overline{X}_{{\text{j}}} + \upbeta_{1} \left( {{\text{X}}_{{{\text{ti}}}} - \overline{X}_{{\text{i}}} } \right) + {\upbeta }_{2} \left( {{\text{X}}_{{{\text{tj}}}} - \overline{X}_{j} } \right) + {\text{r}}_{00i} + {\text{ r}}_{{{\text{00j}}}} + {\text{u}}_{{{\text{0ij}}}} + {\text{e}}_{{{\text{tij}}}}$$

This equation can be split up into its three levels:

Level 1: $${\text{Y}}_{{{\text{tij}}}} = \upbeta_{{0{\text{ij}}}} + \upbeta_{1} \left( {{\text{X}}_{ti} - \overline{X}_{i} } \right) + \upbeta_{2} \left( {{\text{X}}_{{{\text{tj}}}} - \overline{X}_{j} } \right) + {\text{e}}_{{{\text{tij}}}}$$.

Level 2: $$\upbeta_{{{\text{0ij}}}} = \upgamma_{{{\text{00i}}}} + \upgamma_{{{\text{00j}}}} + {\text{u}}_{{{\text{0ij}}}}$$.

Level 3: $$\upgamma_{00i} = \updelta_{000} + \updelta_{001} \overline{X}_{i} + {\text{ r}}_{00i}$$

$$\upgamma_{{{\text{00j}}}} = \updelta_{000} + \updelta_{001} \overline{X}_{{\text{j}}} + {\text{r}}_{{{\text{00j}}}}$$

In this specification ${\text{X}}_{ti}$ refers to a host country characteristic a certain moment in time, while ${\text{X}}_{tj}$ refers to an origin country characteristic a moment in time. ${\overline{\text{X}}}_{i}$ and ${\overline{\text{X}}}_{j}$ refer to the mean of those characteristics per country over time. Specifically, for host countries, X refers to ranking, GDP, and the (natural log of the) total number of students in the host country while for origin countries X only refers to the total population aged 15–24. The mean for X is estimated at level 3 while the difference from the mean is at level 1. None of the other included variables were transformed in this manner, because they either did not vary over time, did not vary between countries, or were not relevant for the host country variables. Finally, at level 1 ${\upbeta }_{{0{\text{ij}}}}$ refers to the intercept of each country pair and ${\text{e}}_{{{\text{tij}}}}$ to the error term. At level 2 ${\upgamma }_{{00{\text{i}}}}$ is the intercept for the host countries while ${\upgamma }_{{00{\text{j}}}}$ is the intercept of the origin countries, and ${\text{u}}_{{0{\text{ij}}}}$ is the error component. At level 3, ${\updelta }_{000}$ refers to the overall intercept ${\updelta }_{001} {\overline{\text{X}}}_{{\text{i}}}$ and ${\updelta }_{001} {\overline{\text{X}}}_{{\text{j}}}$ to the slopes of the mean of the country characteristics, and ${\text{r}}_{{00{\text{i}}}}$ and ${\text{r}}_{{00{\text{j}}}}$ to the random error component for the deviation from the overall intercept. The dyadic and control variables (distance, common border, shared official language, and colonial ties) are not shown in the equation but are placed at level 2.

## Results

### Migration transition theory

**Fig. 1:**
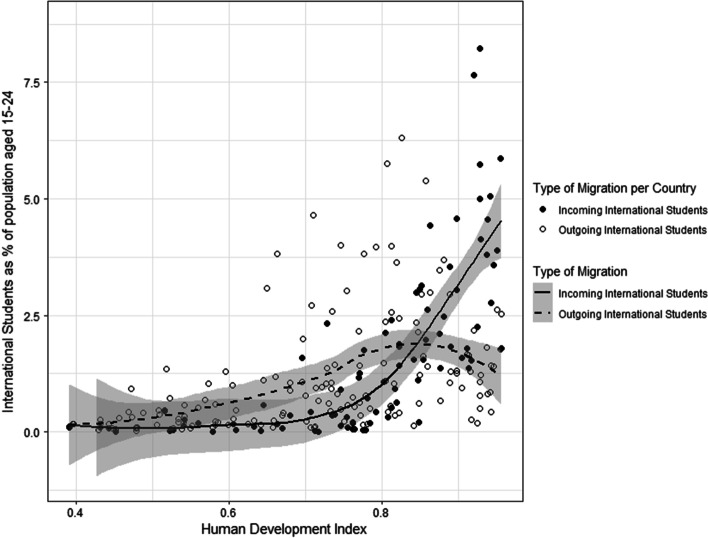
Student migration and human development (2018)

Figure 1 shows the number of incoming and outgoing international students relative to countries’ young population in the year 2018. The dashed line and open circles represent outgoing students, the solid line and closed circles represent incoming students. The lines are estimated as a non-parametric regression based on a loess curve and the dark bands represent confidence intervals. As can be observed, the figure shows an inverted U-shape pattern, as predicted by migration transition theory: when the Human Development Index of countries increases, relatively more students migrate abroad, up until a certain point where the emigration goes down again, though not dropping to the out-migration levels of countries that are low in development—which is also predicted by migration transition theories (De Haas et al., 2020). The immigrant line on the other hand remains fairly flat until countries reach high levels of development upon which they increasingly attract more international students. This reflects the uneven distribution of international students also reported by for example Shields (2013), who found that students tend to mostly move towards a select few highly developed destination countries. As was stated before, we only include the year 2018 for the sake of readability, but in Appendix 2 the same plot can be found for the years 2003, 2008, and 2013. Interestingly, the inverted U-shape only appeared after 2007, before that the relationship between the HDI and outgoing student mobility was much more linear. This could indicate a shift from international student mobility as an elite activity towards one that is more accessible to broader groups of the population, which would be in line with recent observations in the scholarly literature (see e.g. Lipura & Collins, 2020; Brooks & Waters, 2022).

### Descriptive statistics per world region

Table 1 shows an overview of a selection of the most important variables in this study split up into three time periods. The data in the table is based on the same country selection as is used for the regression models. The countries were grouped using the World Bank classification.^11^ It can be seen that all regions have seen an increase in HDI, but some regions, e.g. East Asia and the Pacific, and South Asia, have seen a faster development. This appears to coincide with a faster growing rate of outgoing students compared to other world regions. Regarding incoming and outgoing international students, the results are similar to what we encountered in Fig. 1. That is, the highly developed countries (Europe, Oceania, North America) also have larger percentages of incoming students. Interestingly, the country groupings with the highest rates of outgoing international students are Europe and Central Asia, and the Middle East and North Africa. However, a further inspection of the data revealed that for Europe and Central Asia the outgoing mobility was highest for Eastern Europe and Central Asia which could potentially reflect a move from East to West or from the neighboring countries of Russia to Russia itself. For the middle east and north African countries the high rate of outgoing mobility seems to be much higher in the high-income countries in that region (e.g. Bahrain, Israel, Kuwait, Qatar), which reflects the link between development and outgoing migration. Finally, it can be seen that North America dominates the Shanghai ranking, followed by Oceania, Europe and East-Asia. Finally, the numbers on GDP per capita indicate that most country groupings have become richer, though some (specifically South Asia, and East Asia and the Pacific) have seen a faster growth than the other groupings.

**Table 1:** Descriptive Statistics of HDI, ISM, Ranking, and GDP per World Region (Averages per Country Grouping)

World Bank Region	Human development index			Incoming international students (relative to population aged 15–24)			Outgoing international students (relative to population aged 15–24)		
	2003–2007	2008–2013	2014–2018	2003–2007	2008–2013	2014–2018	2003–2007	2008–2013	2014–2018
East Asia and the Pacific	0.57	0.63	0.66	0.12	0.31	0.47	0.34	0.49	0.76
Europe and Central Asia	0.76	0.79	0.82	0.92	1.29	1.92	1.01	1.32	2.09
Latin America & the Caribbean	0.61	0.65	0.68	0.06	0.08	0.13	0.20	0.22	0.31
Middle East and North Africa	0.56	0.61	0.64	0.49	0.80	1.38	0.67	0.85	1.44
North America	0.85	0.87	0.89	1.34	1.80	2.98	0.50	0.53	0.63
Oceania	0.81	0.92	0.92	4.00	6.65	8.58	0.42	0.33	0.59
South Asia	0.36	0.45	0.50	0.00	0.01	0.02	0.14	0.33	0.68
Sub-Saharan Africa	0.37	0.42	0.45	0.09	0.11	0.13	0.44	0.38	0.39

World Bank Region	Total shanghai ranking Score per country			GDP per capita		
	2003–2007	2008–2013	2014–2018	2003–2007	2008–2013	2014–2018
East Asia and the Pacific	48	47	48	12,135	16,308	19,887
Europe and Central Asia	53	52	63	20,570	25,735	31,301
Latin America and the Caribbean	4	8	8	8976	11,353	14,041
Middle East and North Africa	0	10	8	30,481	42,093	33,680
North America	1741	1667	1545	39,927	45,485	52,837
Oceania	139	176	161	28,580	40,570	43,955
South Asia	5	2	3	3260	4735	8385
Sub-Saharan Africa	2	1	1	4233	4226	4885

### Differences in characteristics of destination countries

Table 2 shows the results of the regression analyses using the between-within models. As was explained earlier, between-within models estimate both differences between countries, and estimate how the change of the predictors over time within countries influence the number of international students that are hosted.

**Table 2:** Regression analyses on the determinants of the choice of destination country of international students, split up by HDI quintile of Origin Countries

	Total	Very low	Low	Medium	High	Very high
Differences between host countries						
Ranking	− 0.02 (0.01)	− 0.09*** (0.02)	0.04* (0.02)	− 0.10*** (0.02)	− 0.01 (0.02)	0.07*** (0.02)
GDP	1.17*** (0.01)	0.74*** (0.02)	1.06*** (0.02)	1.29*** (0.02)	1.56*** (0.02)	1.61*** (0.02)
Change over time within host countries						
Ranking	0.02** (0.01)	0.07*** (0.01)	0.04** (0.01)	0.00 (0.01)	0.02 (0.01)	− 0.02 (0.01)
GDP	0.35*** (0.01)	0.19*** (0.02)	0.42*** (0.02)	0.29*** (0.01)	0.37*** (0.01)	0.34*** (0.01)
Linguistic and colonial ties						
Common language	2.34*** (0.02)	2.46*** (0.05)	2.48*** (0.05)	2.14*** (0.06)	1.52*** (0.07)	0.59*** (0.06)
Colonial past	1.24*** (0.04)	1.53*** (0.12)	1.41*** (0.11)	1.73*** (0.11)	1.30*** (0.08)	0.90*** (0.08)
Control variables						
Student population Host Country (Between)	1.57*** (0.01)	1.70*** (0.02)	1.70*** (0.02)	1.88*** (0.02)	1.55*** (0.02)	1.16*** (0.02)
Student Population Host Country (Within)	0.12*** (0.01)	0.15*** (0.02)	0.13*** (0.01)	0.01 (0.02)	0.12*** (0.02)	− 0.05** (0.02)
Population aged 15–24 Origin country (Between)	1.13*** (0.01)	0.85*** (0.01)	1.37*** (0.01)	1.09*** (0.02)	1.05*** (0.02)	0.83*** (0.01)
Population aged 15–24 Origin country (Within)	0.20*** (0.01)	0.23*** (0.02)	0.02 (0.02)	0.18*** (0.02)	0.34*** (0.02)	0.16*** (0.01)
Distance	− 0.95*** (0.01)	− 0.79*** (0.01)	− 1.19*** (0.02)	− 1.19*** (0.02)	− 1.01*** (0.01)	− 0.78*** (0.01)
Common Border	2.82*** (0.04)	2.93*** (0.12)	1.95*** (0.11)	3.33*** (0.09)	3.02*** (0.06)	2.38*** (0.06)
Number of observations	148,463	30,146	30,286	29,046	29,810	28,932
Number of country Pairs	16,058	3901	4540	4206	4720	3672
Number of host countries	119	116	117	116	114	115
Number of origin countries	145	37	45	41	48	35

*p < 0.05, **p < 0.01, ***p < 0.005. Standard errors are between brackets. All continuous variables are standardized. Included in the regression but not shown in the table is a dummy variable indicating whether a value on the student population of the host country was imputed

The first group of variables, under the heading *Differences Between Host Countries* compares the effects of the variables between countries. For the first variable, ranking, it can be seen that overall countries with higher rankings do not attract more or less international students which contrast previous findings (Van Bouwel & Veugelers, 2013). However, when examining the quintiles we did find effects: students from the low and very high quintile tend to move to countries with high rankings, while students from the very low and medium quintiles are more likely to move to countries with lower rankings. It could be that higher ranked countries attract students from these countries because it is relatively expensive so only students from the richest countries can afford it, while students from lower developed countries settle for cheaper countries. It also connects to the conceptualization of international migration patterns as multi-layered migration hierchies (De Haas et al., 2020: 60), ‘with migrants from middle-income countries often moving to high-income countries, but with middle-income countries attracting migrants from poorer countries in their own right’. The result of the low quintile is more confusing, but it could be that in these countries only students from highly priviliged families are able to study abroad or that it reveals the effect of higher education insitutions in highly developed countries handing out scholarships to students in developing countries. The second group of variables, under the heading *Change Over Time Within Host Countries*, reveals what happens when countries increase in their ranking while ignoring differences between countries. Here it can be seen that countries that have risen in global rankings have also attracted more international students, but only from the least developed countries.

For GDP the between effect show that countries that are on average wealthier than other countries attract more international students and that this effect tends to increase as the origin countries become more developed. Thus while richer countries seem to have a universal appeal, they are most likely to attract students from the highest developed countries which could reveal the existence of global inequalities as high GDP countries also tend to have high costs of living. The within effect of GDP is also positive and significant, but not necessarily stronger for higher or lower developed countries. It does imply that economic growth and incoming student mobility are tied together.

Finally, the effect of having a shared common language and past colonial ties is positive for all quintiles, but becomes stronger for students from lower developed countries. This result is in line with our expectation that students from lower developed countries might be confronted with higher migration costs when deciding to move abroad. In this context, such costs might be lowered by linguistic similarities, and because former colonies and colonizers often have policies in place that facilitate ISM towards the former colonizer.

## Discussion

In this study, we investigated how international student migration flows correlate with countries’ levels of development. Theoretically grounded in migration transition theories, we expected to observe an inverted U-shape, whereby the number of outgoing international students increases when developmental levels increase, until a certain developmental threshold is reached, from which point onwards a decrease in outgoing international student migration would occur. Connecting data from UNESCO Educational Statistics on global student migration flows with the HDI, such an inverted U-curve was indeed observed after 2007. It is likely that this inverted U-shape has started to appear because ISM has started to open up to wider groups of students and is no longer undertaken only by the privileged few (Brooks & Waters, 2022). This suggests that migration transition theories are relevant for explaining the development of international student migration patterns as well.

To probe deeper into this general pattern, we investigated how differences in three destination country characteristics might play a different role in the decision-making process of students from countries with different levels of developments. First, our findings indicated that students from the lowest and medium developed countries were more likely to migrate to lower-ranking destination countries, while students from very highly developed countries most often moved to high-ranking countries. Interestingly, students from the lower developed countries were also more likely to move to high ranking countries, which could be due to a privileged elite that is sending their children to high-ranking countries, such as was the case for China in the 1980s and 1990s (Brooks & Waters, 2011: 47). At the same time, this could also be the result of prestigious higher education institutions handing out scholarships to students in lower developed countries to attract and retain talent. These findings suggest that global international student migration flows are embedded within structural global inequalities, as higher-ranked destination countries are mostly located in countries in the Global North. Theoretically, this finding can be explained by the extra constraints higher ranking countries might place on students’ (and their families') capabilities in the Global South, particularly with regard to finances, as studying in higher ranking countries generally comes together with a significant financial cost, both in terms of tuition fees and subsistence means. In addition, students from the Global South might also face more administrative hurdles to move to higher ranked countries because of immigration and visa legislations. Today, relatively high visa restrictions are observed in Europe and North America for African and Asian citizens, and visa-free travel arrangements are primarily organized in integrated regional blocks such as the EU (De Haas et al., 2019). Together with the financial factor, this might decrease the capabilities of students from the Global South to move to the Global North. Furthermore, we also observed that countries that increase on the rankings become more popular destinations among students originating from countries with lower levels of development. This illustrates the relevance of considering global hierarchies of higher education—in this paper proxied by rankings—in analyses of international student migration, and is also in line with the international student migration literature that builds further upon the human capital perspective (see e.g. Van Bouwel & Veugelers, 2013). If foreign education is an investment decision of individuals, students will prefer to attend a high-quality institution if its higher costs are compensated by higher returns (Van Bouwel & Veugelers, 2013: 173). As such, the ranking of countries in a globally stratified higher education system likely also influences international students’ aspirations in terms of the specific destination countries they would like to go to.

Second, we observed that while all students tend to move to countries with higher GDPs per capita, this effect is more pronounced for students from higher developed countries. On the one hand, this might be related to the financial factor, that is, the more expensive a country is, the more difficult it is for students from less developed countries to bear the costs of international student migration towards that destination. We also found that increases in GDP were associated with increased enrollments in the host countries, but this effect was not stronger for lower or higher developed countries. This could indicate that countries which are growing economically are attractive to all students. Perhaps this is due to international students seeking opportunities in economically emerging countries after graduation, although students do not necessarily inform themselves of employment opportunities before departure (Ginnerskov-Dahlberg, 2021). However, it could also point to the fact that economically emerging countries might put more resources into recruiting international students in order to strengthen their own position on the world’s stage. These findings can thus be related to the rise of regional education hubs and more intra-continental cooperation (see e.g. França & Cairns, 2020 for some examples), which can make it increasingly attractive to move within the Global South. As such, our analysis warns—in line with a recent argument of King and Sondhi (2018: 176–177)—not to oversimplify global international student migration as ‘a dichotomy between the Global North as countries of destination, and those of the Global South as origins of students’, as sizeable movements also take place within lower developed countries and new educational hubs are on the rise (see e.g. Hou & Du, 2020; Kondakci et al., 2018). An example of such an emerging destination country is China as it has seen a large increase in incoming international students, though mostly from developing countries (Yang, 2020) such as other East-Asian or Sub-Saharan African countries (Mulvey, 2021).

Third, we found that a shared common language and colonial history are positively related to international student enrollments, and that these effects are stronger for students from lower developed countries. This implies that it is not just quality of education and wealth of the host country that influences ISM but that linguistic and historical ties matter as well. Students from less developed countries might experience more significant hurdles when choosing a study destination and might therefore choose to study in countries that share familiarities. Students from highly-developed countries on the other hand might experience more opportunities in where they can study. It also implies that the relationship between previously colonized countries is still fairly one-directional with students being more likely to move towards former colonizers instead of the other way around. In Europe, important examples of this are France and Portugal who draw in many students from Africa and Brazil respectively. The fact that language ties also have a positive effect shows that students might prefer similar countries (e.g. students from the UK moving to the US) but it could also point to historical ties. Russia, for example, has long acted as a core country (from a world systems perspective) by recruiting many students from its neighboring countries, many of which were former satellites. However, it should also be kept in mind that it is not just students making these kinds of considerations; countries can also exploit cultural ties to more effectively recruit students. Furthermore, new ISM patterns related to structural dependencies could also be emerging, such as China’s involvement in sub-Saharan Africa where young people are encouraged to study in China in order to strengthen political ties.

Overall, our study adds to the existing literature by demonstrating on a large scale that developmental differences between countries can explain which destination country international students primarily intend to move to. This complements the studies of, for example, Kondakci (2011), Wei (2013), and Perkins and Neumayer (2014), who all found important differences between students from developed and developing countries. However, these studies generally consider a developed/developing countries dichotomy while this study has shown that the empirical reality is more nuanced and non-linear. As countries reach higher stages of development the capabilities of its (student) population expand, but their aspirations also change which influences the destinations they choose. The choice of destination countries is also not static, but changes as countries of origin and destination develop. In sum, our study indicates that migration transition theories offer a useful framework for interpreting the migration patterns of international students, in particular the increasing diversification of student migration flows (Choudaha & Van Mol, 2021; Kondakci et al., 2018). Through the lens of migration transition theory this diversification could be explained by considering that, as capabilities are generally increasing across the world, more young people are able to follow up on their aspirations to study abroad. However, top destination countries might still be inaccessible leading them to choose for countries with closer geographical, cultural, and linguistic proximity.

There are some limitations to this study that are worth mentioning. Because of the scope of this paper, focusing on global student migration flows, some variables that can be expected to influence these flows are not included. For example, tuition fees of host countries are likely an important consideration for students. Although some international comparable data is available on tuition fees, we do not possess such data for all of the relevant countries and for all of the relevant years, making it an unsuitable variable for the empirical analysis of this paper. Some countries in this sample did not have data available for all the years. The most important examples are that there is no data on how many international students were hosted by China and Germany (before 2013), though both countries did have data available on the number of outgoing students.

## Conclusion

Our study showed that migration transition theories could be a useful starting point to investigate ISM. As countries develop, the number of outgoing students first tends to increase and then decrease, while the number of incoming students tends to show a more consistent increase. In line with previous findings, we found that GDP, university ranking, and linguistic/colonial ties were positively associated with increased enrollments of international students. However, we also found that the strength of these effects differed depending on the level of development of origin countries.

These findings also have some policy implications as traditional destination countries should keep in mind changing numbers and demographics of the international students they host. As China is developing its economy and educational system, the number of outgoing international students is expected to decrease while emerging countries such as Vietnam, India, and Nigeria are projected to become the major source countries (Choudaha, 2017). At the same time traditional destination countries should count on more competition from emerging destination countries. Finally, as the demographic structure of international student population is changing, institutions should take care to ensure the well-being of international students as different cultures have different ways of coping with the stress of being an international student (Akhtar & Kroener-Herwig, 2019).

Future research could further investigate how differences in the make-up of international student populations impact migration patterns. For example, it could be worthwhile to study how the COVID-19 pandemic impacted ISM differently countries of different levels of development. Migration transition theories offer a fruitful lens, and our study has made progress using this theory, but there is still much that needs to be investigated. Micro-level studies can shed further light on the exact capabilities and aspirations that are experienced by international students, but it is also important to conduct more multi-level studies that can take into account individual preferences and how these interact with contextual characteristics. Not only differences in development might be relevant, cultural and gender differences could potentially also play a role as could differences in personality and preferences. As Lipura and Collins (2020, p. 12) state ‘a more critical take on ISM’s diversification would pay attention to the intersections of class, national, gender, age, and other factors in shaping student mobility and achievements. Only when the existence of stratified inequalities between these intersections are recognized can mobilities, especially in contemporary forms, be understood holistically’.

## Data Availability

All of the data and other materials are available upon request.

## Appendix 1: List of countries included in this study

### Host countries included in the regression analyses

Afghanistan, Albania, Argentina, Armenia, Australia, Austria, Azerbaijan, Bahrain, Belarus, Belgium, Benin, Bhutan, Bosnia and Herzegovina, Botswana, Brazil, Bulgaria, Burkina Faso, Burundi, Cape Verde, Cambodia, Cameroon, Canada, Central African Republic, Chad, Chile, Colombia, Congo, Costa Rica, Croatia, Czech Republic, Democratic Republic of the Congo, Denmark, Dominican Republic, Ecuador, Egypt, El Salvador, Estonia, Eswatini, Fiji, Finland, France, Georgia, Germany, Ghana, Greece, Guinea, Guyana, Honduras, Hungary, India, Indonesia, Iran (Islamic Republic of), Ireland, Israel, Italy, Ivory Coast, Japan, Jordan, Kazakhstan, Kenya, Kuwait, Kyrgyzstan, Lao People's Democratic Republic, Latvia, Lesotho, Lithuania, Madagascar, Malaysia, Mali, Mauritania, Mexico, Mongolia, Morocco, Mozambique, Myanmar, Namibia, Nepal, the Netherlands, New Zealand, Niger, North Macedonia, Norway, Oman, Pakistan, Palestine, Peru, Philippines, Poland, Portugal, Qatar, Republic of Korea, Republic of Moldova, Romania, Russian Federation, Rwanda, Saudi Arabia, Senegal, Serbia, Slovakia, Slovenia, South Africa, Spain, Sri Lanka, Sweden, Switzerland, Tajikistan, Thailand, Tunisia, Turkey, Turkmenistan, Ukraine, United Arab Emirates, United Kingdom, United Republic of Tanzania, United States, Uzbekistan, Venezuela (Bolivarian Republic of), Vietnam, Zimbabwe.

### Origin countries included in the regression analyses

Afghanistan, Albania, Algeria, Angola, Argentina, Armenia, Australia, Austria, Azerbaijan, Bahrain, Bangladesh, Belarus, Belgium, Benin, Bhutan, Bosnia and Herzegovina, Botswana, Brazil, Bulgaria, Burkina Faso, Burundi, Cambodia, Cameroon, Canada, Cape Verde, Chad, Chile, China, Colombia, Costa Rica, Croatia, Cuba, Czech Republic, Denmark, Djibouti, Dominican Republic, Ecuador, Egypt, El Salvador, Eritrea, Estonia, Eswatini, Ethiopia, Fiji, Finland, France, Gabon, Gambia, Georgia, Germany, Ghana, Greece, Guatemala, Guinea, Guinea-Bissau, Guyana, Honduras, Hungary, India, Indonesia, Iran (Islamic Republic of), Iraq, Ireland, Israel, Italy, Ivory Coast, Jamaica, Japan, Jordan, Kazakhstan, Kenya, Kuwait, Kyrgyzstan, Lao People's Democratic Republic, Latvia, Lebanon, Lesotho, Liberia, Libya, Lithuania, Luxembourg, Madagascar, Malawi, Malaysia, Mali, Mauritania, Mexico, Mongolia, Morocco, Mozambique, Myanmar, Namibia, Nepal, the Netherlands, New Zealand, Niger, Nigeria, North Macedonia, Norway, Oman, Pakistan, Palestine, Panama, Paraguay, Peru, Philippines, Poland, Portugal, Qatar, Romania, Rwanda, Saudi Arabia, Senegal, Serbia, Singapore, Slovakia, Slovenia, South Africa, Spain, Sri Lanka, Sweden, Switzerland, Tajikistan, Thailand, the Central African Republic, the Congo Republic, the Democratic Republic of the Congo, the Republic of Korea, the Republic of Moldova, the Russian Federation, Ukraine, United Arab Emirates, United Kingdom, United Republic of Tanzania, United States, Yemen, Togo, Tunisia, Turkey, Turkmenistan, Uganda, Uruguay, Uzbekistan, Venezuela (Bolivarian Republic of), Vietnam, Zambia, Zimbabwe.

## Abbreviation

ISM: International student mobility

## Footnotes

1 Authors own calculations, based on data retrieved from UNESCO.

2 Which measures only degree mobility see: http://uis.unesco.org/sites/default/files/documents/uoe-data-collection-manual-2020-en.pdf.

3 http://data.uis.unesco.org/.

4 Notable missing host countries are Bangladesh, Ethiopia, Nigeria, Pakistan, the Philippines, and Tanzania. With these included the sample would contain 90% of the world’s population.

5 We chose to only show 2018 because showing all years together made the plot difficult to read. However, when we computed the plots for the other years nearly identical patterns emerged.

6 Notable missing host countries are China, Bangladesh, Nigeria, and Ethiopia. If these were included the sample would account for 93.2% of the world’s population.

7 We also ran the analyses with the total student population of the origin country instead of the population aged 15–24 of the origin country. However, this did not substantially change the results.

8 http://www.shanghairanking.com/.

9 https://data.worldbank.org/indicator/NY.GDP.PCAP.CD.

10 http://cepii.fr/CEPII/en/welcome.asp.

11 https://datahelpdesk.worldbank.org/knowledgebase/articles/906519-world-bank-country-and-lending-groups.
